# Enabling Telemedicine From the System-Level Perspective: Scoping Review

**DOI:** 10.2196/65932

**Published:** 2025-03-05

**Authors:** Xuezhu Li, Lifeng Huang, Hui Zhang, Zhanming Liang

**Affiliations:** 1 School of Public Health Sun Yat-sen University Guangzhou China; 2 College of Public Health, Medical and Veterinary Science James Cook University Townsville, Queensland Australia

**Keywords:** telemedicine, telehealth, digital health, success factors, challenges

## Abstract

**Background:**

Telemedicine is a strategy for providing health care services remotely that improves service accessibility. Telemedicine has attracted growing research interest in the past 10 years, including systematic reviews that synthesize evidence to share experiences and enhance knowledge. However, most of the published systematic reviews have focused on synthesizing evidence from studies on telemedicine at the organizational level. A collected understanding of factors on the system level that influence the successful implementation and adoption of telemedicine needs to be developed, especially in regional and rural areas.

**Objective:**

This scoping review aims to explore key success factors and challenges that influence the implementation and adoption of telemedicine at the system level, particularly in regional and rural areas.

**Methods:**

This scoping review was conducted in accordance with the framework by Arksey and O’Malley and reported using the PRISMA-ScR (Preferred Reporting Items for Systematic Reviews and Meta-Analyses Extension for Scoping Reviews). A total of 5 databases (CINAHL, Cochrane, Medline, Ovid, and Scopus) were searched for research articles published in English between January 2010 and 2023, using the established inclusion criteria.

**Results:**

Of the 10,691 papers identified, 89 were included in this review, including 16 (17.98%) studies conducted in regional and rural areas and 13 (14.61%) in metropolitan areas. Another 13 (14.61%) studies were conducted in both metropolitan areas and regional and rural areas. Overall, 6 categories with more than 70 key success factors, including system-level requirements (n=13, 18.40%), economic considerations and funding (n=6, 8.70%), technological requirements (n=6, 8.70%), organizational requirements (n=19, 27.54%), understanding and supporting clinicians (n=12, 17.39%), and understanding and improving patients’ perceptions (n=13, 18.84%), were identified. Additionally, 5 categories containing over 50 challenges, including those related to system levels (n=11, 23.91%), technological requirements (n=6, 13.04%), organizational requirements (n=13, 28.26%), clinicians (n=10, 21.74%), and patients (n=6, 13.04%), were identified. Among the identified factors, 11 (9.57%) were specific to regional and rural areas.

**Conclusions:**

This scoping review confirms that the successful implementation of telemedicine requires collective efforts at both the system and organizational levels, including coordination and collaboration across different regions and organizations. It underscores the importance of establishing a national network that enhances public awareness of telemedicine and clarity in payment and benefit distribution models and strengthens data security protection measures. The review also highlights the necessity of addressing infrastructural deficiencies, including internet connectivity in regional and rural areas, and suggests the implementation of targeted incentives and support measures. The required collective efforts are detailed in the proposed framework that promotes popularizing telemedicine, enhancing the overall quality and efficiency of health care services, and achieving broader health equity.

## Introduction

### Background

Telemedicine enables medical professionals to provide diagnosis and treatment to patients from different locations by using phone or video calls. It is regarded as a strategy for addressing many challenges facing health care service provision, particularly for improving the accessibility of services provided remotely [1]. The World Health Organization defines telemedicine as “the delivery of health care services, where distance is a critical factor, by all health care professionals using information and communication technologies for the exchange of valid information for diagnosis, treatment and prevention of disease and injuries, research and evaluation, and for the continuing education of health care providers, all in the interests of advancing the health of individuals and their communities” [2]. This definition has been commonly applied in the 10 years since it was first published [3-6]. Telemedicine is one of the main tools in the current development of health systems, with significant potential for diagnosing, treating, and preventing diseases [7]. Different from the broad definition of telehealth, which covers both clinical and nonclinical services including training and continuing medical education for practitioners, telemedicine solely refers to remote clinical services [8]. However, both telemedicine and telehealth are core components of eHealth, a concept that includes all health care practices supported by electronic processes such as information and communication technologies (ICTs) [9,10].

Telemedicine can be implemented in the following 3 models: televisit, telemonitoring, and tele-expertise [11]. Televisits occur between doctors and patients through phone calls, video conferencing software, or secure email systems [11]. Telemonitoring involves using personal health technologies to collect, transmit, evaluate, and communicate patients’ personal health data from outside hospitals or clinical departments (eg, patients’ homes) and relay them to health care providers [12]. Tele-expertise allows health care professionals to seek medical opinions and guidance from, and share patient data with, experts via phone, video conferencing software, or secure email [13]. According to the care delivery modalities, telemedicine can be divided into synchronous and asynchronous telemedicine [14]. Synchronous telemedicine requires a real-time 2-way interaction between the doctor and patient [1] or between doctors [15]. In asynchronous telemedicine, clinical data elements, such as medical reports, images, and video recordings, are stored and transmitted for later evaluation [16]. Patients send photos of lesions, radiological scans, and lab results to doctors, who respond later in the same manner [17]. Based on these implementation and delivery methods, telemedicine is widely used in medical consultations, patient follow-ups, specialist consultations, drug prescription and delivery, teletriage and screening, and telerehabilitation, among other services [18].

The existing evidence indicates that telemedicine can bring multiple benefits. For patients, the use of telemedicine increases access to care [19], improves care outcomes [20], reduces costs [21], and leads to high patient satisfaction [22,23]. For health care providers, telemedicine saves a significant amount of time and costs [24] and reduces travel time [25]. Health care providers also show high satisfaction with telemedicine [26]. Given the numerous benefits of telemedicine, it is important to understand and identify its key enabling factors and the specific challenges in implementing it.

### Key Success Factors

The existing research has focused on factors that enable the implementation and adoption of telemedicine services, including individual, organizational, technological, and economic factors. One individual enabling factor is doctor training and skill-building, which can mitigate the knowledge and skill gaps among health care personnel, enabling them to use telemedicine more easily [27]. Additionally, patients’ satisfaction with telemedicine [28] and their positive opinion of digital devices [29] are also key individual factors for effective telemedicine implementation. At the organizational level, coordination between different health care levels and integrating telemedicine into clinicians’ workflows [27] can enable the successful implementation of telemedicine services. At the technological level, the availability of technical support, good network coverage, user-friendly applications, and privacy protection are important [30]. Furthermore, in low- and middle-income countries, economic factors may include funding telemedicine projects to purchase equipment and software and pay for mobile services [30]. In high-income countries and regions, establishing a financial framework for telemedicine, financial benefits, and cost savings is a key economic factor [27].

### Challenges Facing Telemedicine Implementation and Adoption

On the other hand, barriers to the adoption and implementation of telemedicine have been identified in the literature, including technology and equipment, patient privacy, user attitudes, and reimbursement policies. Common technology and equipment–related barriers include the lack of telemedicine equipment, inability to connect to the internet [31], and lack of access to or comfort with the necessary technology for a video visit [32]. User attitudes toward telemedicine could result in resistance to using telemedicine among both health care providers [33] and patients [34]. Evidence also indicates that the lack of reimbursement for telemedicine services [35] hinders the adoption and implementation of telemedicine.

### Efforts in Synthesizing Evidence on Telemedicine

To further understand the current state of the research on telemedicine, we systematically retrieved 305 scoping reviews, systematic reviews, and meta-analysis papers on telemedicine published between 2010 and 2023 from the Scopus database. The search and review of the 305 review articles confirmed that existing studies mainly focused on the following four aspects: (1) the application of telemedicine before and after COVID-19 (n=63, 20.66%), particularly in specific diseases or populations; (2) evaluating the effectiveness of telemedicine use (n=72, 23.61%), such as reducing patient mortality and improving patient quality of life; (3) the economic evaluation of telemedicine (n=109, 35.74%), such as cost-effectiveness and cost-utility analyses; and (4) examining factors influencing the implementation and adoption of telemedicine (n=43, 14.10%).

Among these scoping reviews, systematic reviews, and meta-analysis papers, the majority focused on synthesizing evidence from studies on telemedicine at the organizational level. Only a few papers synthesized information from studies focusing on understanding factors on the societal level or funding, policy, and regulation (system-level) factors influencing the successful implementation and adoption of telemedicine. System-level factors, beyond the boundaries of individual organizations, specifically refer to the national structures and policy environments, funding, and investment in infrastructure required to deliver telemedicine services, a process that involves collaborative efforts across multiple organizations [36]. Furthermore, a thorough understanding of the challenges facing telemedicine implementation and adoption in regional and rural areas is limited. Therefore, a scoping review was conducted to answer the following questions:

(1) What are the key factors that can influence the implementation and adoption of telemedicine at the system level, particularly in regional and rural areas?

(2) What are the key challenges facing the implementation and adoption of telemedicine at the system level, particularly in regional and rural areas?

## Methods

### Overview

**The systematic review was conducted** following the 5-step process described by Arksey and O’Malley [37], chosen for the exploratory and descriptive nature of our study objectives. The keywords and abstract screening, conducted by the authors XL and LH, only included studies in the health context. Authors XL and LH worked on the full-text review and data extraction together, in consideration of their lack of experience in conducting the systematic literature review and data extraction. Author ZL reviewed abstracts that received contradictory assessment outcomes from XL and LH. ZL also performed full-text reviews on the articles when required. A thematic analysis of the factors that influence the implementation of telemedicine was performed by contracted research officer LK. The Covidence systematic literature review platform [38] was used to perform both the abstract and full-text screening.

On December 16, 2023, a literature search was performed in the following 5 databases: CINAHL, Cochrane, Medline, Ovid, and Scopus. Table 1 details the 4 key concepts with associating keywords that were used to conduct the keyword search. Each search strategy includes keywords from concept 1 and either concept 2, 3, or 4. The key concepts were connected using the conjunction “AND,” and the keywords under the same key concept were connected using the logical operator “OR.” Search terms used were not restricted to the title only. They were found within the title, abstract, or keywords.

**Table 1:** Keywords and key concepts.

Concept 1	Concept 2	Concept 3	Concept 4
Telemedicine	Factors	Design	Location
Telehealth Tele-health Tele-care Telemedicine Tele-medicine Remote clinical service Tele-critical care (same concept use OR)	Barriers Challenge Difficulty Facilitator Factors Failure Obstacles Success	Application Execution Financing Funding Implementation Model Operation Regulation Strategy	District Nonmetropolitan Regional Remote Rural

### Inclusion and Exclusion Criteria

Papers that met all the following criteria were included in the data extraction: (1) published in English in or after 2010; (2) published in a peer-reviewed journal; and (3) research findings that are relevant to 1 or 2 of the research questions for the review. Papers that did not meet all the aforementioned criteria were excluded from the final data extraction.

### Data extraction

An Excel (Microsoft Corp) spreadsheet was set up for data extraction with the following categories: (1) year of publication; (2) country where the study was conducted; (3) purpose of the paper; (4) study design; (5) methods for data collection; (6) study population and sector; (7) target population; (8) sample size; (9) response rate; (10) metro or regional/rural location; and (11) factors that influence the adoption or implementation of telemedicine.

### Data Synthesis

The data synthesis focused on key factors and challenges and involved a descriptive summary of the included studies. Guided by Braun and Clarke’s thematic analysis [39], key themes were identified by examining data patterns and grouping similar concepts. A comparative analysis was performed to explore differences in findings between regional/rural and metropolitan areas. The results were synthesized to identify commonalities and discrepancies in the literature, addressing identified challenges that guided the development of a framework for implementing and adopting telemedicine. To ensure the accuracy of the analytical process, a codebook with descriptive meanings of each theme was generated and discussed between the authors to refine codes and enhance the depth of the analysis.

### Critical Appraisal

In line with the scoping review framework, we did not conduct a critical appraisal of the papers [37].

## Results

### Study Selection

**Figure 1:**
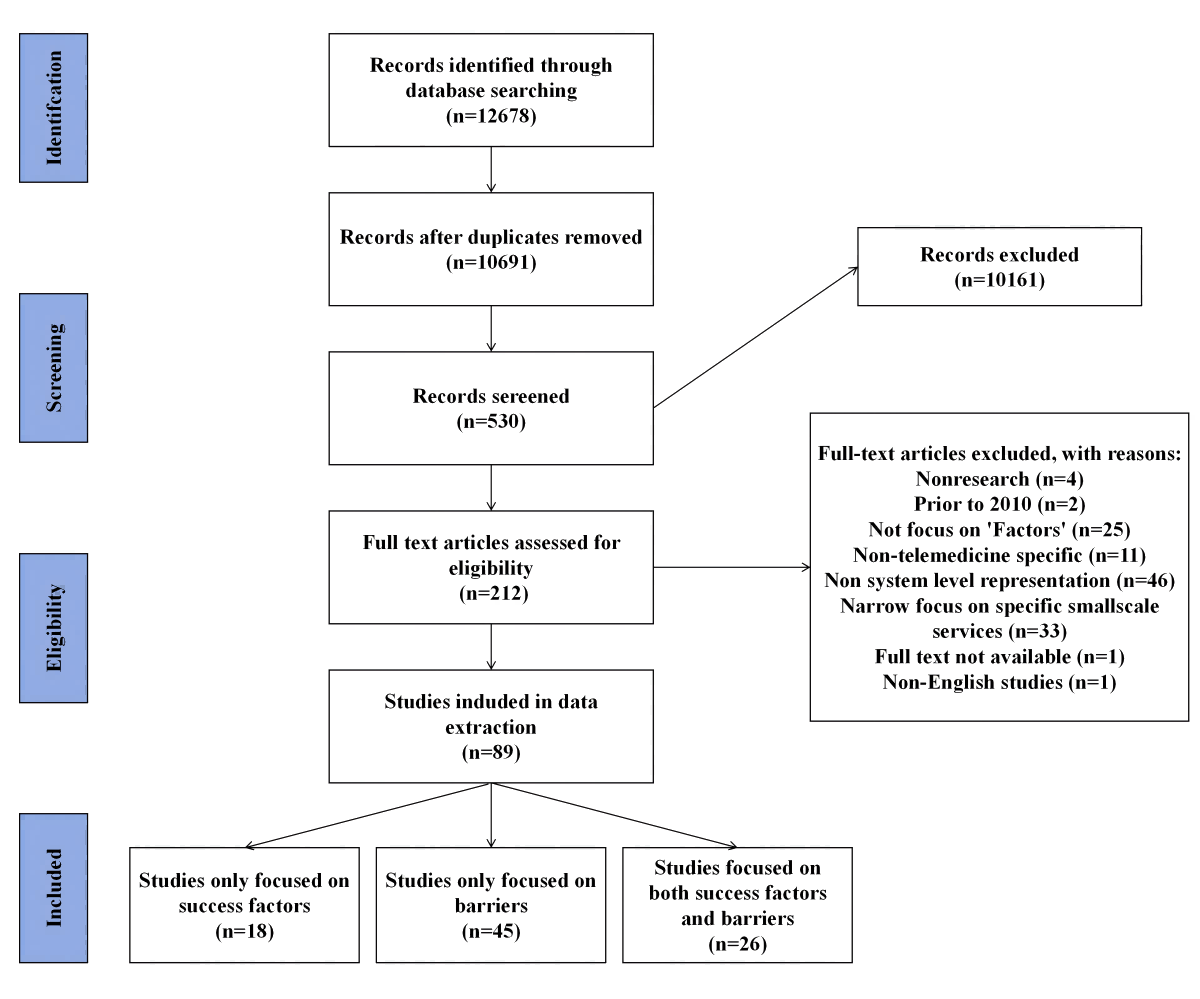
PRISMA (Preferred Reporting Items for Systematic Reviews and Meta-Analyses) flowchart.

This study was reported according to the PRISMA-ScR (Preferred Reporting Items for Systematic Reviews and Meta-Analyses Extension for Scoping Reviews) guidelines [40]. The completed PRISMA-ScR checklist is available in Multimedia Appendix 1. The PRISMA flowchart (Figure 1) details the review process and search results. The initial key concept and keyword search found 10,691 potentially relevant articles from the 4 databases. Title screening reduced the number to 530 papers, which were uploaded to Covidence [38] for abstract screening. After the abstract screening, 212 articles were included in the full-text review. The full-text review confirmed the inclusion of 89 articles for data extraction. Multimedia Appendix 2 contains a description of the 89 articles.

### Study Location and Year of Publication

Publication dates ranged from 2010 to 2023 (Multimedia Appendix 3). A total of 66 (74.16%) studies were published between 2020 and 2023. Among them, 4 (4.49%) studies were conducted in multiple countries in the following continents: the Middle East, South America and Europe, Sub-Saharan Africa, and North America. A total of 28 (31.46%) studies were conducted in the United States, 10 (11.24%) in Australia, 5 (5.62%) in China, 3 (3.37%) in Canada, 3 (3.37%) in Ethiopia, 3 (3.37%) in Germany, 3 (3.37%) in the United Kingdom, 2 (2.25%) in Egypt, and 2 (2.25%) in Uganda. Only 1 (1.12%) study was conducted on a global scale, which included 75 countries. Another 25 (28.10%) studies were conducted in 25 different countries. Of the 89 studies, 13 (14.61%) were conducted in metropolitan areas and 16 (17.98%) in regional and rural areas. Another 13 (14.61%) studies were conducted in both metropolitan areas and regional and rural areas.

### Sectors and Target Population

A total of 21 (23.60%) studies were conducted in multiple sectors (hospitals, primary health care, nursing homes, aged care, health authorities, and so on). On the other hand, 55 (61.80%) studies were single-sector studies, including 37 (41.57%) studies in hospitals, 14 (15.73) in primary health care, 2 (2.25%) in community health services, and 2 (2.25%) in mental health. Another 13 (14.61%) studies did not specify the sectors in which they were conducted.

Of the 89 studies, 31 (34.83%) focused on multiple target groups including patients, clinicians, managers, and service providers. The other 58 (65.17%) studies focused on single target groups, including 27 (30.34%) on clinicians, 18 (20.22%) on service providers, 6 (6.74%) on other users (eg, technicians, decision-makers, and medical students), 5 (5.62%) on patients, and 2 (2.25%) on managers.

### Methods Used to Identify Factors

A total of 46 (51.69%) and 35 (39.33%) studies adopted quantitative and qualitative approaches, respectively. Among the studies, 10 (11.24%) adopted a mixed methods approach combining both quantitative and qualitative methods. The most common methods for data collection were surveys (n=43, 48.31%), interviews (n=24, 26.97%), and focus group discussions (n=7, 7.87%).

### Key Success Factors

Among the identified articles, 44 (49.44%) discussed factors important to the implementation and adoption of telemedicine. More than 70 factors were mentioned in different papers. Based on the similarities and differences, these factors were organized into 6 different categories, as detailed in Textbox 1. The factors were placed into 4 categories—individual, organizational, technological, and economic. Individual factors were subcategorized based on their relevance to clinicians and patients. A category of system-level requirements was also created to accommodate the health system–level factors identified in this review. In total, the review identified 16 (17.98%) studies that focused on regional, rural, and/or remote areas. Factors identified in these studies were compared with the factors associated with metropolitan areas. For each included source of evidence, the detailed data that were charted are displayed in Multimedia Appendix 4.

Factors important for the success of telemedicine (listed in alphabetical order). The * symbol indicates factors that are common to both metropolitan and nonmetropolitan areas. Factors that are specific to nonmetropolitan areas are italicized. ICT: information and communications technology

**System-level requirements**

- Established performance requirements and monitoring mechanism
- Improved security and reliability*
- Improvement of digital and health literacy
- National Health ICT index
- Policy and guidelines for the use of telemedicine
- Promotion of telemedicine (to both the workforce and the community)
- Provision of incentives
- Remuneration for service providers
- Robust telehealth program design*
- Telehealth equipment and software applications
- Workforce development
- Additional support and infrastructure investment in rural and remote areas
- Addressing unmet health care needs

**Economic considerations and funding**

- Cost and benefits sharing between providers and stakeholders
- Financial support and remuneration
- Health insurance coverage and arrangements
- Prior consideration of economic feasibility
- Provision of equipment grants
- Sustained funding allocation (beyond the grant period)

**Technological requirements**

- Data security and protection
- Efficient and functional network and information-sharing
- Improved ICT infrastructure and support
- Telehealth equipment and software applications
- Up-to-date computers, software, and telecommunication devices
- User friend software and system

**Organizational requirements**

- Engaging staff in designing the telemedicine goals and processes
- Established performance indicators that measure the benefits of telemedicine to patients, clinicians, and organizations
- Governance and policy
- ICT infrastructure and support
- Improved awareness of the benefits of telemedicine across organizations
- Leadership and management support
- Needs assessment and preparation
- Ongoing monitoring and modification of staff workload
- Patient education and support
- Promotion of telemedicine to both patients and key stakeholders
- Provision of incentives
- Provision of technical support
- Resources and support
- Simplified, standardized, and transparent processes
- Staff capability and competence
- Stakeholder engagement support
- Utilization of existing expertise
- Well-established quality improvement and monitoring processes
- Visible staff champions

**Understanding and supporting clinicians**

- Being flexible and adaptive to change*
- Digital skills*
- Feeling empowered and efforts are recognized
- Manageable workload and appropriate transition
- Perceived benefits of telemedicine for themselves, patients, and the organization *
- Positive perception and attitudes toward telemedicine*
- Positive teamwork*
- Receiving regular information and updates*
- After-hours staff support
- Encouraged safer clinical practices
- Increased access to relevant resources
- Perceived patient benefits and quality of care

**Understanding and improving patients’ perceptions**

- Access to required telecommunication devices*
- Established confidence and trust in clinicians and the organization
- Increased awareness of telemedicine and its benefits to themselves
- Positive perception and attitudes
- Preexisting relationship with clinicians and organization
- Prior exposure to telemedicine
- Support in improving digital and health literacy
- Timely and accessible support and troubleshooting
- Transparent feedback system*
- Good internet access
- Improved patient-provider communication
- Improved patient experiences and outcomes
- Previous positive experience with telemedicine

### Major Challenges Facing Telemedicine Implementation and Adoption

A total of 71 (79.78%) articles identified articles discussed challenges facing the implementation and adoption of telemedicine. More than 50 challenges were mentioned in different papers. These challenges (Textbox 2) were placed into the same categories as those created for the success factors.

**Challenges facing telemedicine (listed in alphabetical order). ICT: information and communications technology.**

**System-level**

- Absence of nationwide integrated telemedicine framework
- Absence of regulatory framework
- Absence of coordination of telemedicine at the national level
- Inadequate efforts in addressing network and data security concerns
- Lack of funding to support organization readiness
- Lack of insurance coverage
- Lack of performance monitoring
- Lack of standardized and specific guidelines for the protection of patient privacy and confidentiality
- Social and cultural concerns
- Unclear reimbursement mechanism
- Unclear or confusing financial arrangements and funding and insurance model

**Technological requirements**

- High cost of equipment
- Lack of ICT infrastructure
- Lack of integration with other digital/online platforms
- Not user-friendly
- Poor internet access
- Unclear data processing procedure and techniques

**Organizational requirements**

- Concerns about meeting legal requirements
- High staff turnover and burnout
- Human resources issues, such as high staff turnover and increased training demands
- Inadequate information and data sharing
- Inadequate budgeting and funding to meet increased costs, such as investment in technology upgrades
- Increased administration and human resource support requirements
- Lack of investment in training and upskilling prior to the implementation of telemedicine
- Legal concerns (eg, potential lawsuits because of patient complaints)
- Negative effects on quality of care (eg, long wait time, poor clinician-patient interaction, reduction of patient safety, and lack of physical examination)
- Patient scheduling
- Staff workload issues
- Unclear governance and policy direction
- Unclear performance and outcome measures

**Clinicians**

- Concerns about patient confidentiality
- Inadequate digital literacy
- Lack of confidence and trust from patients
- Lack of confidence in patient consent and choices
- Lack of familiarity with new equipment, software, and devices
- Patient scheduling
- Resistance from patients
- Resistance from team members
- Responsibility for data security and protection
- Unmanageable workload

**Patients**

- Affordability of digital devices
- Increased isolation due to the reduced interaction with clinicians and health care organizations
- Language barriers
- Patient resistance due to various factors that were not addressed, such as reduced contact time with clinicians and unclear consultation mechanisms and feedback systems
- Perceived decreased access to care
- Poor internet access

## Discussion

### Principal Findings

This study fills a knowledge gap by confirming key system-level factors that are critical to the successful implementation and adoption of telemedicine, as well as factors specific to nonmetropolitan areas. Particularly, the study reinforced the importance of 4 success factors that have not been discussed in published systematic review papers. The first factor is the development of a national health ICT index, a comprehensive measure of a country’s ICT capabilities, which is crucial for the large-scale implementation of telemedicine [41]. The index includes national ICT infrastructure and access, ICT usage levels, and ICT skills [41]. The second factor is the provision of additional support and investment in building necessary infrastructure in rural and remote areas to enhance telemedicine adoption in these regions [42-44]. The third factor is the need for targeted efforts to raise awareness and promote the benefits of telemedicine among both health care professionals and patients, addressing misconceptions and facilitating adoption [45,46]. The fourth factor is investment in innovation and robust design in telemedicine programs, which can enhance efficiency and sustainability [47-50]. This study also suggests the necessity of developing a nationwide integrated telemedicine framework to improve coordination across national, regional, and local telemedicine initiatives and ensure that national and local policies and strategies are aligned [51]. The factors identified in this scoping review are common to health care organizations, rather than problems specific to individual organizations, and hence may need to be addressed at the system level.

### Framework for the Implementation and Adoption of Telemedicine

**Figure 2:**
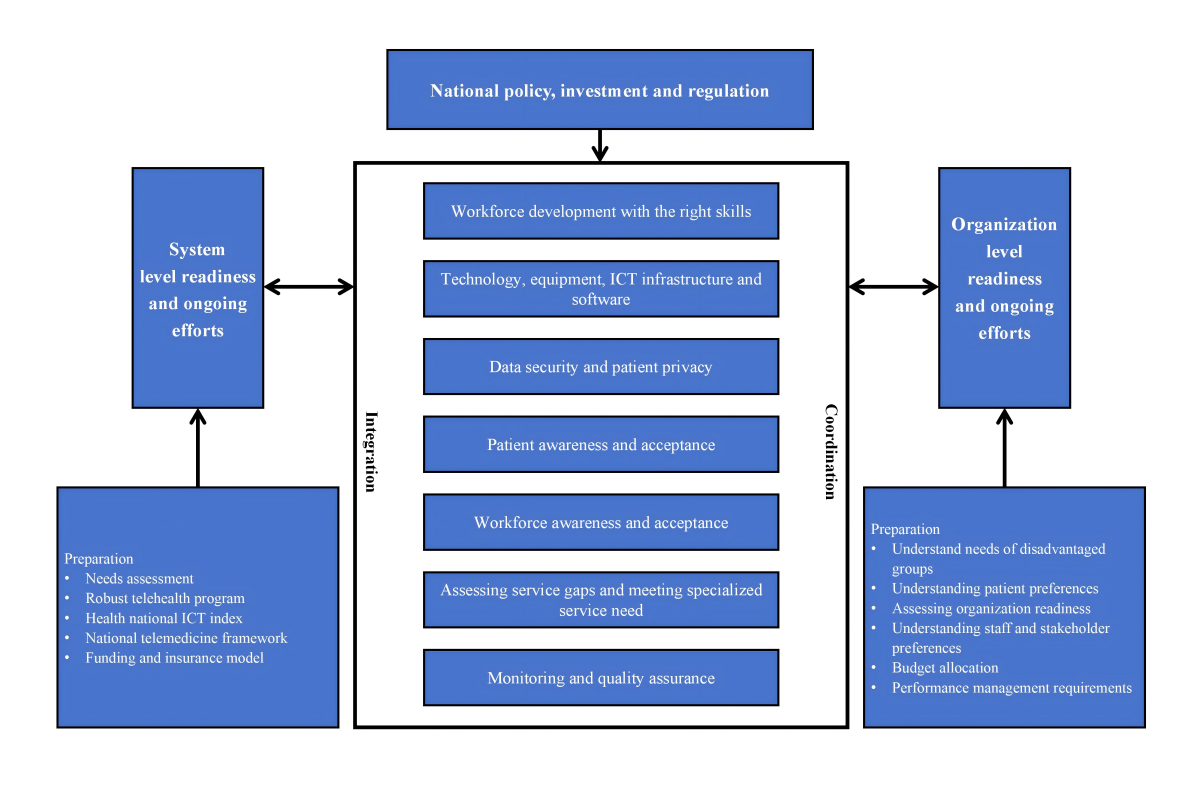
Framework for the successful implementation of telemedicine. ICT: information and communication technology.

This scoping review confirms that a successful implementation of telemedicine requires collective efforts at both the system and organizational levels. At the national level, policies and regulations to set the direction, quality expectation, and boundary of telemedicine service provision are necessary. Additionally, investments in improving infrastructure and system-level readiness and addressing potential barriers that may undermine the benefits of telemedicine are required [41]. These efforts can be categorized into 7 core areas, as illustrated in Figure 2—a framework that guides the implementation and adoption of telemedicine proposed by our study. The framework highlights the changes and ongoing efforts required within health systems and health care organizations to help realize the benefits of telemedicine. Continuous improvements to enhance the maturity of health care organizations provide a foundation for innovation and future system and organizational transformations. It is also critical to achieve strategic alignment across the system, between the system and organizations, and across the individual organization internally. The success of telemedicine is a system matter rather than a silo organization business [51].

Specifically, the successful implementation of telemedicine necessitates assessment at both system and organizational levels of their respective readiness, as well as the efforts required [36,52]. For a system as complex as health care, different sectors and organizations are interconnected; hence, the implementation of telemedicine is a system-level matter rather than an individual health care organization’s sole business [53]. Therefore, the successful implementation of telemedicine requires the adoption of a system integration approach. Organizational requirements; directions; and system-side funding, policy, and regulation must be aligned to enable cooperation and coordination across organizations [36]. Coordination implies the necessity for intraorganizational and interorganizational collaboration and coordination to ensure that key factors that facilitate the successful implementation of telemedicine are addressed or attended to [54].

Furthermore, common factors that impact the implementation of telemedicine cannot be considered in isolation due to the dynamic, bidirectional relationship between factors at the system and organizational levels. Enhancing common factors may influence the readiness and maturity of systems and organizations, providing a foundation for innovation and thereby altering the preparation and effort required for future system-level and organization-level transformations.

As mentioned in the Introduction, there are factors localized to health care organizations that impact the implementation of telemedicine, such as ICT infrastructure, internet connectivity, technical support systems [55], reimbursement, and data security [56]. This scoping review confirms that we must consider these factors at the system level, as they cannot be resolved by a single organization. Hence, support and efforts at both the organization and system level are required. The successful implementation of telemedicine typically requires strong team leadership, suitable training, flexibility, the use of cost-effective and straightforward systems, and implementation within a defined legal and regulatory framework [57]. Addressing organization-level barriers at the system level enables organizations to implement telemedicine successfully.

### Improving Awareness, Understanding, and Readiness

One of the key factors emerging in recent studies is resistance to the utilization of telemedicine by both health care professionals and patients, which is common across health care organizations and countries [58-61]. This is partly attributed to health professionals and patients’ lack of understanding of telemedicine’s benefits [62], patients’ fear of losing face-to-face interaction with doctors [63], and the difficulties encountered in utilizing telemedicine [64]. Efforts are required to improve the health workforce and patients’ and carers’ understanding of the important role and inevitable trend of telemedicine in improving the quality of care. It requires broader government actions to raise public awareness [45] and improve health and digital literacy [44]. Evaluating health systems and organizations’ readiness for implementing telemedicine is a critical step that requires adequate preparation, planning, support, and coordination across the health system. This may include developing policies that broadly guide telemedicine design, implementation, and utilization [45,65,66]. Studies also suggest the necessity of offering incentives and financial support to health care organizations in improving infrastructure and enhancing training for health care workers and patients [47,67]. This systemic approach ensures that the unique circumstances of each organization are considered, facilitating a more effective and widespread adoption of telemedicine.

### Financial Arrangements, Funding, and Insurance Model

Health insurance coverage, financial arrangements, funding mechanisms, and health insurance models were discussed in 23 (25.84%) identified papers. Additionally, concerns about lower reimbursement rates provided for telemedicine services compared to face-to-face visits, and inappropriate reimbursement strategies were raised [68]. Although some of the factors were discussed in a previous review paper that focused on the organizational level [69], this scoping review explores these factors from a system-level perspective. Unclear or confusing financial arrangements and payment models were identified as key obstacles to the successful adoption of telemedicine [59], as the development of payment models has not kept pace with the rapid expansion of telemedicine.

Internationally, the traditional fee-for-service payment system is still predominantly used to reimburse or pay for telemedicine services [11]. As a result, clinical providers may alter the way services are provided to ensure they receive adequate reimbursement. For instance, some previously free medical services offered by health professionals were modified for new names such as audio-only telemedicine visits or e-visits, to be billed [70]. This non–user-friendly service model discourages patients from accessing services, hindering the development and expansion of telemedicine. Systematically designed payment policies and payment models that support and adequately reimburse service providers are crucial for the continuous development and adoption of telemedicine [71]. The examined studies suggested exploring hybrid models that combine capitation payment and fee-for-service payment [11], bundled payment models [72,73], and other models that are superior to the traditional fee-for-service model to promote telemedicine adoption.

To design a feasible and equitable funding model for telemedicine, mechanisms that support cost and benefit sharing among stakeholders must first be established. For example, a study in Norway [51] shows that telemedicine is primarily supported by internal organizational funds or funded through national or regional research and innovation projects, lacking sustainable long-term financing channels, which affects the continuous development and scaling of telemedicine projects. A feasible and equitable cost-sharing mechanism requires partnership between governments, health care institutions, and other stakeholders, making telemedicine broadly accessible and sustainable. Additionally, mechanisms that enable fair benefit distribution and provide incentives to stakeholders are critical. For example, in the United States, specialized services are often provided by more advanced and well-equipped medical centers or hospitals to patients initially treated and referred by primary care providers. Under the current funding model, the primary care advanced medical centers or hospitals may receive greater economic returns. Medicare only pays a fixed telemedicine service facility fee to the initiating party—primary care institutions—while paying the providing party 100% of the equivalent offline service fees [74]. Additionally, general practitioners may experience increased workloads without proportional financial gain due to inadequate compensation mechanisms [75]. Ultimately, primary care providers, being the least favorable party for benefit distribution, lack the motivation to support telemedicine. The distribution of benefits in telemedicine is thus a complex issue that requires comprehensive consideration and the gradual development of mechanisms to promote equitable redistribution among all stakeholders [51].

### Network and Data Security

Previously published systematic reviews confirmed that the stability and security of the telemedicine network, as well as patient and service data, are among the key concerns of telemedicine adoption at health care organizations [56,57]. This review, based on 16 (17.98%) identified papers, confirmed that it is a key systemic factor that hinders the success of telemedicine and must be promptly addressed in and across health care organizations and the health system. Network concerns are related to not only problems of internet availability and connectivity [43,76,77] but also the absence of an integrated nationwide telemedicine network that supports the adoption of telemedicine [51]. Internet availability and connectivity concerns refer to internet availability [43], network latency [76], and poor network connections [77]. These issues pose significant technical challenges to the adoption of telemedicine. On the other hand, the lack of coordination of telemedicine initiatives at the national, regional, and local levels has resulted in the fragmentation of the health care sector, hindering the construction of a nationwide integrated telemedicine network [51].

To build a nationwide integrated telemedicine network, institutions must coordinate and align their strategic plans to national strategies and policies, while ensuring patient quality and safety [51]. Concerns about data security were identified among both health professionals and patients [78]. These concerns are related to low digital literacy levels, measures adapted to enable the secure use of information, and adopted communication technologies [79]. Patient concerns about data security can lead to resistance to using telemedicine, highlighting the need for clinical standards, certifications for service providers and institutions offering telemedicine services, and robust legal frameworks to protect user and institutional data security [7].

### Telemedicine in Regional and Rural Areas

Considering health disparities, geographical constraints, and limitations on infrastructure in regional and rural areas, telemedicine is an important initiative that should address the health care needs of the local population and improve service provision by clinicians, including addressing the challenges of workforce shortages [80,81]. Telemedicine presents an opportunity to alleviate the imbalance between health care demand and supply in nonmetropolitan areas by meeting patients’ urgent needs for high-quality health care services and addressing disparities in health care resource allocation, diagnostic and treatment capabilities, and quality between urban and rural areas [82]. For patients living in rural and remote areas, telemedicine is becoming critical to enhancing access to health care specialists [83] and high-quality health care services, such as timely e-consultations and tailored treatment plans [84] from their homes, through telemedicine platforms. On the other hand, telemedicine allows health care providers to receive support and expert advice online, thus directly improving the capacity of health professionals to deliver quality patient care [81]. To fully leverage the potential of telemedicine, comprehensive strategies and substantial investments in infrastructure, along with training for health professionals who provide or receive services via telemedicine platforms, are required [85].

One significant challenge in regional and rural areas is the lack of infrastructure enabling the smooth adoption and implementation of telemedicine. Poor access and unstable internet service have limited the coverage and effectiveness of telemedicine initiatives. Therefore, prioritizing functional broadband access with a sufficient download speed that meets the requirements of telemedicine in rural areas is an essential government/system-level investment [85,86]. Creating a community broadband network may be a good example [86]. It is worth noting that even when broadband is available, the affordability of broadband usage can be a barrier for rural populations [86]. Therefore, government/system-level investments must include relevant policy support and incentives that enable affordable broadband service provision, such as subsidies for broadband deployment and usage, in underserved rural areas [87].

Telemedicine is a core strategy for improving health service availability and accessibility in regional, rural, and remote areas. To maximize its intended benefits, telemedicine must be designed to address the specific health care needs of the servicing areas [43,81] and enhance the support to clinical staff for safer and more effective clinical practices [42], such as remote expert consultation and after-hours staff support [80]. Studies also confirmed the importance of addressing the skepticism and fear of how losing face-to-face interactions between patients and clinicians may affect the quality of care received [88]. Hence, specific strategies should be developed to enhance patient-provider communication in the process of telemedicine implementation and adoption [81].

### Limitations

First, this study only searched for English articles, and studies in other languages were excluded, which may be a potential limitation. Second, due to the inability to access and analyze unpublished articles, some important factors might not have been included. Third, only studies about telemedicine related to clinical services were included in this review, and some studies may have been overlooked.

### Conclusions

This study reveals the key system-level factors for the successful implementation of telemedicine in health care systems and provides an in-depth insight into factors specific to regional and rural areas. The results show that the widespread implementation of telemedicine is constrained by multifaceted factors, including economy, technology, organization, and individual factors. This study underscores the importance of establishing a national network enhancing public awareness of telemedicine, ensuring clarity in payment and benefit distribution models, and strengthening data security protection measures. In addition, for regional and rural areas, this study highlights the necessity for addressing infrastructural deficiencies, particularly in terms of internet connectivity, and suggests the implementation of targeted incentives and support measures. In summary, this study provides valuable insights for policy makers, health care administrators, and health care providers by highlighting the importance of coordination and collaboration across different regions and organizations. Through these efforts as detailed in the proposed framework, it is possible to promote the popularization of telemedicine, enhance the overall quality and efficiency of health care services, and achieve broader health equity.

## Appendix

Multimedia Appendix 1 PRISMA-ScR (Preferred Reporting Items for Systematic Reviews and Meta-Analyses Extension for Scoping Reviews) checklist.

Multimedia Appendix 2 Description of the 89 articles.

Multimedia Appendix 3 Year of publication and number of studies.

Multimedia Appendix 4 Data extraction.

## Abbreviations

ICT: information and communication technology

## Data Availability

The data used and analyzed during the current study are available from the corresponding author upon reasonable request.
