# Supplementary material for: Enabling Telemedicine From the System-Level Perspective: Scoping Review
Source: J Med Internet Res. 2025 Mar 5;27:e65932. doi: 10.2196/65932 (PMC11923472; doi:10.2196/65932)
Supplement: Multimedia Appendix 2 [file jmir_v27i1e65932_app2.docx]

**Multimedia Appendix 2 Description of the 89 articles.**

| Study | Country | Year of publication | Sectors | Target population | Methods for data collection |
| --- | --- | --- | --- | --- | --- |
| Alboraie et al. (2022) | Egypt | 2022 | hospitals | Clinicians, managers and other users | Quantitative |
| Allan et al. (2021) | Australia | 2021 | hospitals | Clinicians, managers and other users | Qualitative |
| Klee et al. (2023) | US | 2023 | primary health care | patients and providers | Quantitative |
| Alaboudi et al. (2016) | Saudi Arabia (KSA) | 2016 | multiple sectors, including all types of Healthcare Facilities | managers | Mixed-methods |
| Alajlani and Clarke (2013) | Jordan and Syria | 2013 | hospitals, primary healthcare | Clinicians, patients and other users | Qualitative |
| Alami et al. (2017) | Norway | 2017 | multiple sectors, including hospitals, regional and national health authorities and universities | providers | Qualitative |
| Albarrak et al. (2021) | Saudi Arabia | 2021 | hospitals | clinicians | Quantitative |
| Alboraie et al. (2021) | Egypt | 2021 | hospitals | other users | Quantitative |
| Alkmim et al. (2015) | Brazil | 2015 | primary health care | clinicians | Quantitative |
| Al Meslamani et al. (2022) | the United  Arab Emirates (UAE) | 2022 | not-specific | other users | Quantitative |
| Antonacci et al. (2023) | Italy | 2023 | multiple sectors including local health authorities, public hospital enterprises, IRCCS, nursing homes, and other public and private healthcare | clinician, managers | Quantitative |
| Assaye et al. (2023) | Ethiopia | 2023 | hospitals | clinicians | Quantitative |
| Baker et al. (2022) | America | 2022 | hospitals | providers | Quantitative |
| Balchander et al. (2022) | America | 2022 | primary health care | Providers | Qualitative |
| Banbury et al. (2023) | Australia | 2023 | hospitals | providers | Qualitative |
| Banbury et al. (2023) | Australia | 2023 | hospitals, primary healthcare | patient and other users | Mixed-methods |
| Bello et al. (2017) | Canada | 2017 | multiple sectors including hospital, primary healthcare, and others | patients and providers | Qualitative |
| Berry et al. (2023) | USA | 2023 | primary health care | Qualitative data: providers | Qualitative |
| Bhatta et al. (2015) | Nepal | 2015 | hospitals | providers | Qualitative |
| Bhatia et al. (2022) | USA | 2022 | multiple sectors including hospital, aged care, and primary health care | patients | Mixed-methods |
| Clure et al. (2023) | USA | 2023 | not-specific | other users | Quantitative |
| Dünnebeil et al. (2012) | Germany | 2012 | hospitals, primary healthcare | clinicians | Quantitative |
| Garcia-Huidobro et al. (2020) | Chile | 2020 | hospitals, primary healthcare | Clinicians, patients | Mixed-methods |
| Moroz et al. (2020) | Canada | 2020 | multiple sectors including provincial and territorial governments, national organisations, and others | patients and providers | Qualitative |
| Casillas et al. (2022) | USA | 2022 | multiple sectors including hospital, primary healthcare, community health center | providers | Qualitative |
| Choi et al. (2019) | South Korea | 2019 | hospitals | patients, providers, and others | Qualitative |
| Etz et al. (2023) | USA | 2023 | primary health care | clinicians | Quantitative |
| Hansen et al. (2022) | USA | 2022 | hospitals | providers | Qualitative |
| Howland et al. (2021) | USA | 2021 | mental health | clinicians | Qualitative |
| Isautier et al. (2020) | Australia | 2020 | not-specific | other users | Mixed-methods |
| Kabukye et al. (2023) | Uganda | 2023 | hospitals | Clinicians, patients, managers | Qualitative |
| Kane and Gillis (2018) | USA | 2018 | hospitals, primary health care | clinicians | Quantitative |
| Kiberu et al. (2019) | Uganda | 2019 | hospitals | patients and providers | Mixed-methods |
| Le Bras et al. (2023) | France | 2023 | not-specific | quantitative data: users  qualitative data: clinicians, managers | Qualitative |
| Li et al. (2022) | China | 2022 | hospitals | providers | Quantitative |
| Lingg and Lütschg (2020) | Switzerland | 2020 | multiple sectors including hospitals, medical device industry, health sector associations, government- and research-related bodies | providers | Qualitative |
| Ma et al. (2022) | China | 2022 | hospitals | clinicians | Quantitative |
| Moffatt and Eley (2011) | Australia | 2011 | not-specific | providers | Qualitative |
| Murphy et al. (2021) | UK | 2021 | primary health care | clinician, managers | Qualitative |
| Newman et al. (2016) | Australia | 2016 | mental health | providers | Qualitative |
| Rabinowitz et al. (2023) | USA | 2023 | primary health care | clinician, managers | Qualitative |
| Romero et al. (2018) | Spain | 2018 | primary health care | clinicians | Quantitative |
| Shi et al. (2023) | China | 2023 | hospitals | clinicians | Quantitative |
| Shiferaw et al. (2021) | Ethiopia | 2021 | multiple sectors including operation centers, hospitals, health facilities | providers | Quantitative |
| Theis et al. (2023) | USA | 2023 | multiple sectors including hospital, primary healthcare, government-related bodies | providers | Qualitative |
| Vimalananda et al. (2023) | USA | 2023 | not-specific | patients | Quantitative |
| Walczak et al. (2022) | Poland | 2022 | primary health care | clinicians | Quantitative |
| Wherton et al. (2021) | Scotland | 2021 | not-specific | patients and providers | Mixed-methods |
| Yu-tong et al. (2022) | China | 2022 | hospitals | clinicians | Quantitative |
| Zobair et al. (2019) | Bangladesh | 2019 | hospitals | patients | Quantitative |
| Boyle et al. (2023) | USA | 2023 | hospitals | managers | Quantitative |
| Chen et al. (2021) | USA | 2021 | hospitals | providers (hospitals) | Quantitative |
| Cui et al. (2020) | China | 2020 | hospitals | clinician, managers | Quantitative |
| Duclos et al. (2017) | Burkina Faso | 2017 | primary health care | Clinicians and other users | Qualitative |
| Indria et al. (2020) | Indonesia | 2020 | primary health care | clinicians | Qualitative |
| Nyoni et al. (2023) | USA | 2023 | hospitals | Clinicians, managers | Qualitative |
| Price-Haywood et al. (2023) | USA | 2023 | primary health care | Clinicians, patients | Qualitative |
| Vakkalanka et al. (2022) | USA | 2022 | hospitals | Clinicians, patients, managers | Qualitative |
| Zachrison et al. (2023) | USA | 2023 | hospitals | clinicians | Quantitative |
| Diel et al. (2023) | Germany | 2023 | hospitals | clinicians | Quantitative |
| Kang et al. (2022) | UK | 2022 | hospitals | clinicians | Mixed-methods |
| Nduka et al. (2023) | Nigeria | 2023 | Community health services | Clinicians, patients | Quantitative |
| Onsongo et al. (2023) | Kenya | 2023 | hospitals, primary healthcare | clinicians | Quantitative |
| Riew et al. (2021) | internation | 2021 | hospitals | clinicians | Quantitative |
| Saigí-Rubió et al. (2014) | Bolivia, Spain, Colombia | 2014 | hospitals, primary healthcare | clinicians | Quantitative |
| Wade et al. (2012) | Australia | 2012 | system-level policy | clinician, managers | Qualitative |
| Crotty et al. (2021) | USA | 2021 | hospitals, primary healthcare | Clinicians, patients | Quantitative |
| Elkefi and Layeb (2023) | USA | 2023 | not-specific | patient and other users | Mixed-methods |
| Gutierrez et al. (2020) | USA | 2020 | hospitals | clinicians | Qualitative |
| Johnston et al. (2020) | Australia | 2020 | primary health care | providers | Qualitative |
| Khasawneh et al. (2023) | Jordan | 2023 | not-specific | other users | Quantitative |
| Kissi et al. (2020) | The Republic of Ghana | 2020 | hospitals | clinician, managers | Quantitative |
| Lee et al. (2018) | subSaharan African countries and Organization for Economic Cooperation and Development countries | 2018 | system-level policy | other users | Quantitative |
| Luciano et al. (2020) | the United States and Brazil | 2020 | not-specific | Clinicians and other users | Quantitative |
| Martin et al. (2012) | USA | 2012 | hospitals | clinician, managers | Quantitative |
| Ly et al. (2017) | Senegal | 2017 | hospitals | clinician, managers | Qualitative |
| Mathew et al. (2023) | Australia | 2023 | Community health services | providers | Qualitative |
| Muehlensiepen et al. (2021) | Germany | 2021 | hospitals, primary healthcare | clinicians | Quantitative |
| Ray et al. (2017) | USA | 2017 | hospitals | clinician, managers | Qualitative |
| Renner et al. (2023) | Canada | 2023 | hospitals, primary healthcare | clinicians | Quantitative |
| Těšinová et al. (2023) | Czech Republic | 2023 | multiple sectors including hospital, medical society, pharmacist society | patients and providers | Qualitative |
| Wubante et al. (2022) | Ethiopia | 2022 | hospitals | clinicians | Quantitative |
| Zachrison et al. (2020) | USA | 2020 | hospitals | clinicians | Quantitative |
| Zailani et al. (2014) | Malaysia | 2014 | hospitals | clinicians | Quantitative |
| Zhang and Zaman (2020) | Pakistan | 2020 | hospitals | patients | Quantitative |
| Zulman et al. (2019) | USA | 2019 | not-specific | providers | Mixed-methods |
| Forbes et al. (2023) | USA | 2023 | hospitals | patients | Quantitative |
| Ashley et al. (2023) | Australia | 2023 | primary health care | Clinicians | Qualitative |
| Courtney et al. (2021) | UK | 2021 | hospitals | clinicians | Qualitative |

**Reference**

Al Meslamani, A. Z., R. Aldulaymi, H. El Sharu, Z. Alwarawrah, O. M. Ibrahim and N. Al Mazrouei (2022). "The patterns and determinants of telemedicine use during the COVID-19 crisis: A nationwide study." Journal of the American Pharmacists Association: JAPhA 62(6): 1778-1785.

Alaboudi, A., A. Atkins, B. Sharp, A. Balkhair, M. Alzahrani and T. Sunbul (2016). "Barriers and challenges in adopting Saudi telemedicine network: The perceptions of decision makers of healthcare facilities in Saudi Arabia." Journal of Infection and Public Health 9(6): 725-733.

Alajlani, M. and M. Clarke (2013). "Effect of culture on acceptance of telemedicine in Middle Eastern countries: case study of Jordan and Syria." Telemedicine journal and e-health : the official journal of the American Telemedicine Association 19(4): 305-311.

Alami, H., M. P. Gagnon, R. Wootton, J. P. Fortin and P. Zanaboni (2017). "Exploring factors associated with the uneven utilization of telemedicine in Norway: a mixed methods study." BMC Medical Informatics & Decision Making 17: 1-15.

Albarrak, A. I., R. Mohammed, N. Almarshoud, L. Almujalli, R. Aljaeed, S. Altuwaijiri and T. Albohairy (2021). "Assessment of physician's knowledge, perception and willingness of telemedicine in Riyadh region, Saudi Arabia." Journal of Infection and Public Health 14(1): 97-102.

Alboraie, M., M. Abdalgaber, N. Youssef, I. Moaz, N. Abdeen, H. M. Abosheaishaa, M. T. Shokry, F. El-Raey, S. S. Asfour, W. A. Abdeldayem, A. A. Hassan, E. E. M. O. Mahran, M. Tag-Adeen, O. Elshaarawy, M. I. Radwan, A. Altonbary and Y. Fouad (2022). "Healthcare Providers' Perspective about the Use of Telemedicine in Egypt: A National Survey." International Journal of Telemedicine and Applications 2022.

Alboraie, M., M. A. Allam, N. Youssef, M. Abdalgaber, F. El-Raey, N. Abdeen, R. E. Mahdy, O. Elshaarawy, A. Elgebaly, T. Haydara, S. Abd-Elsalam, Y. A. Nassar, H. Shabana and S. Zaky (2021). "Knowledge, Applicability, and Barriers of Telemedicine in Egypt: A National Survey." International Journal of Telemedicine and Applications 2021.

Alkmim, M. B. M., M. S. Marcolino, R. M. Figueira, L. Sousa, M. S. Nunes, C. S. Cardoso and A. L. Ribeiro (2015). "Factors Associated with the Use of a Teleconsultation System in Brazilian Primary Care." Telemedicine and e-Health 21(6): 473-483.

Allan, J., E. Webster, B. Chambers and S. Nott (2021). "“This is streets ahead of what we used to do”: staff perceptions of virtual clinical pharmacy services in rural and remote Australian hospitals." BMC Health Services Research 21(1).

Antonacci, G., E. Benevento, S. Bonavitacola, L. Cannavacciuolo, E. Foglia, G. Fusi, E. Garagiola, C. Ponsiglione and A. Stefanini (2023). "Healthcare professional and manager perceptions on drivers, benefits, and challenges of telemedicine: results from a cross-sectional survey in the Italian NHS." BMC health services research 23(1): 1115.

Ashley, C., A. Williams, S. Dennis, S. McInnes, N. A. Zwar, M. Morgan and E. Halcomb (2023). "Telehealth‘s future in Australian primary health care: a qualitative study exploring lessons learnt from the COVID-19pandemic." BJGP Open 7(2).

Assaye, B. T., M. Belachew, A. Worku, S. Birhanu, A. Sisay, M. Kassaw and H. Mekonen (2023). "Perception towards the implementation of telemedicine during COVID-19 pandemic: a cross-sectional study." BMC health services research 23(1): 967.

Baker, M. C., S. L. King, N. Sikka, E. A. Krupinski, S. A. Shipman and M. Haberman (2022). "Trends in Adoption and Maturation of Telehealth Programs at Teaching Hospitals and Health Systems." Telemedicine journal and e-health : the official journal of the American Telemedicine Association 28(4): 517-525.

Balchander, D., C. I. Cabrera, B. Zack, S. Porter, J. Sunshine and B. D'Anza (2022). "Assessing Telehealth Through the Lens of the Provider: Considerations for the Post-COVID-19 Era." Telemedicine journal and e-health : the official journal of the American Telemedicine Association 28(12): 1806-1816.

Banbury, A., A. C. Smith, A. Mehrotra, M. Page and L. J. Caffery (2023). "A comparison study between metropolitan and rural hospital-based telehealth activity to inform adoption and expansion." Journal of telemedicine and telecare 29(7): 540-551.

Banbury, A., M. Taylor, L. Caffery, C. Der Vartanian, H. Haydon, R. Mendis, K. Ng and A. Smith (2023). "Consumers’ experiences, preferences, and perceptions of effectiveness in using telehealth for cancer care in Australia." Asia-Pacific Journal of Clinical Oncology 19(6): 752-761.

Bello, A. K., A. E. Molzahn, L. P. Girard, M. A. Osman, I. G. Okpechi, J. Glassford, S. Thompson, E. Keely, C. Liddy, B. Manns, K. Jinda, S. Klarenbach, B. Hemmelgarn and M. Tonelli (2017). "Patient and provider perspectives on the design and implementation of an electronic consultation system for kidney care delivery in Canada: A focus group study." BMJ Open 7(3).

Berry, C. A., L. Kwok, M. Gofine, M. Kaufman, D. A. Williams, K. Terlizzi, M. Alvaro and C. J. Neighbors (2023). "Utilization and Staff Perspectives on an On-Demand Telemedicine Model for People with Intellectual and Developmental Disabilities Who Reside in Certified Group Residences." Telemedicine Reports 4(1): 204-214.

Bhatia, R., E. Gilliam, G. Aliberti, A. Pinheiro, M. Karamourtopoulos, R. B. Davis, L. DesRochers and M. A. Schonberg (2022). "Older adults' perspectives on primary care telemedicine during the COVID‐19 pandemic." Journal of the American Geriatrics Society 70(12): 3480-3492.

Bhatta, R., K. Aryal and G. Ellingsen (2015). "Opportunities and Challenges of a Rural-telemedicine Program in Nepal." Journal of Nepal Health Research Council 13(30): 149-153.

Boyle, T., K. Boggs, J. Gao, M. McMahon, R. Bedenbaugh, L. Schmidt, K. S. Zachrison, E. Goralnick, P. Biddinger and C. A. Camargo (2023). "Hospital-Level Implementation Barriers, Facilitators, and Willingness to Use a New Regional Disaster Teleconsultation System: Cross-Sectional Survey Study." JMIR public health and surveillance 9: e44164.

Casillas, A., C. Valdovinos, E. Wang, A. Abhat, C. Mendez, G. Gutierrez, J. Portz, A. Brown and C. R. Lyles (2022). "Perspectives from leadership and frontline staff on telehealth transitions in the Los Angeles safety net during the COVID-19 pandemic and beyond." Frontiers in Digital Health 4.

Chen, J., A. Amaize and D. Barath (2021). "Evaluating Telehealth Adoption and Related Barriers Among Hospitals Located in Rural and Urban Areas." Journal of Rural Health 37(4): 801-811.

Choi, W. S., J. Park, J. Y. B. Choi and J.-S. Yang (2019). "Stakeholders' resistance to telemedicine with focus on physicians: Utilizing the Delphi technique." Journal of Telemedicine & Telecare 25(6): 378-385.

Clure, C., J. Sheeder, S. Teal and R. Cohen (2023). "Telemedicine to improve reproductive health care for rural Coloradans: Perceptions of interest and access." Journal of Rural Health 39(1): 172-178.

Courtney, E., D. Blackburn and M. Reuber (2021). "Neurologists' perceptions of utilising tele-neurology to practice remotely during the COVID-19 pandemic." Patient Education & Counseling 104(3): 452-459.

Crotty, B. H., N. Hyun, A. Polovneff, Y. Dong, M. C. Decker, N. Mortensen, J. M. Holt, A. N. Winn, P. W. Laud and M. M. Somai (2021). "Analysis of Clinician and Patient Factors and Completion of Telemedicine Appointments Using Video." JAMA Network Open 4(11): e2132917-e2132917.

Cui, F., Q. Ma, X. He, Y. Zhai, J. Zhao, B. Chen, D. Sun, J. Shi, M. Cao and Z. Wang (2020). "Implementation and Application of Telemedicine in China: Cross-Sectional Study." JMIR mHealth and uHealth 8(10): e18426.

Diel, S., E. Doctor, R. Reith, C. Buck and T. Eymann (2023). "Examining supporting and constraining factors of physicians' acceptance of telemedical online consultations: a survey study." BMC health services research 23(1): 1128.

Duclos, V., M. Yé, K. Moubassira, H. Sanou, N. H. Sawadogo, G. Bibeau and A. Sié (2017). "Situating mobile health: A qualitative study of mHealth expectations in the rural health district of Nouna, Burkina Faso." Health Research Policy and Systems 15.

Dünnebeil, S., A. Sunyaev, I. Blohm, J. M. Leimeister and H. Krcmar (2012). "Determinants of physicians' technology acceptance for e-health in ambulatory care." International Journal of Medical Informatics 81(11): 746-760.

Elkefi, S. and S. Layeb (2023). "Telemedicine's future in the post-Covid-19 era, benefits, and challenges: a mixed-method crosssectional study." Behaviour & Information Technology 42(15): 2639-2653.

Etz, R. S., C. A. Solid, M. M. Gonzalez, E. Britton, K. C. Stange and S. R. Reves (2023). "Telemedicine in Primary Care: Lessons Learned About Implementing Health Care Innovations During the COVID-19 Pandemic." Annals of family medicine 21(4): 297-304.

Forbes, R. C., W. R. Johnson, N. D'Souza, A. Dreher, S. A. Rega, I. D. Feurer and B. P. Concepcion (2023). "Disparities in telemedicine utilization among kidney transplant patients during the COVID-19 pandemic: Barriers and opportunities." Clinical Transplantation.

Garcia-Huidobro, D., S. Rivera, S. V. Chang, P. Bravo and D. Capurro (2020). "System-wide accelerated implementation of telemedicine in response to COVID-19: Mixed methods evaluation." Journal of Medical Internet Research 22(10).

Gutierrez, J., J. Moeckli, N. McAdams and P. J. Kaboli (2020). "Perceptions of Telehospitalist Services to Address Staffing Needs in Rural and Low Complexity Hospitals in the Veterans Health Administration." Journal of Rural Health 36(3): 355-359.

Hansen, R. N., B. M. Saour, B. Serafini, B. Hannaford, L. Kim, T. Kohno, R. James, W. Monsky and S. P. Seslar (2022). "Opportunities and Barriers to Rural Telerobotic Surgical Health Care in 2021: Report and Research Agenda from a Stakeholder Workshop." Telemedicine journal and e-health : the official journal of the American Telemedicine Association 28(7): 1050-1057.

Howland, M., M. Tennant, D. J. Bowen, A. M. Bauer, J. C. Fortney, J. M. Pyne, J. Shore and J. M. Cerimele (2021). "Psychiatrist and Psychologist Experiences with Telehealth and Remote Collaborative Care in Primary Care: A Qualitative Study." The Journal of rural health : official journal of the American Rural Health Association and the National Rural Health Care Association 37(4): 780-787.

Indria, D., M. Alajlani, H. Sf. Fraser and H. S. F. Fraser (2020). "Clinicians perceptions of a telemedicine system: a mixed method study of Makassar City, Indonesia." BMC Medical Informatics & Decision Making 20(1): N.PAG-N.PAG.

Isautier, J. M. J., T. Copp, J. Ayre, E. Cvejic, G. Meyerowitz-Katz, C. Batcup, C. Bonner, R. Dodd, B. Nickel, K. Pickles, S. Cornell, T. Dakin and K. J. McCaffery (2020). "People's experiences and satisfaction with telehealth during the COVID-19 pandemic in Australia: Cross-sectional survey study." Journal of Medical Internet Research 22(12).

Johnston, K., D. Smith, R. Preston, R. Evans, K. Carlisle, J. Lengren, H. Naess, E. Phillips, G. Shephard, L. Lydiard, D. Lattimore and S. Larkins (2020). ""From the technology came the idea": safe implementation and operation of a high quality teleradiology model increasing access to timely breast cancer assessment services for women in rural Australia." BMC Health Services Research 20(1): 1-12.

Kabukye, J. K., J. Namugga, C. J. Mpamani, A. Katumba, J. Nakatumba-Nabende, H. Nabuuma, S. S. Musoke, E. Nankya, E. Soomre, C. Nakisige and J. Orem (2023). "Implementing Smartphone-Based Telemedicine for Cervical Cancer Screening in Uganda: Qualitative Study of Stakeholders’ Perceptions." Journal of Medical Internet Research 25(1).

Kane, C. K. and K. Gillis (2018). "The Use Of Telemedicine By Physicians: Still The Exception Rather Than The Rule." Health Affairs 37(12): 1923-1930.

Kang, S., L. Raja, D. A. Sim, P. B. M. Thomas and D. G. Ezra (2022). "Telemedicine in oculoplastic and adnexal surgery: Clinicians' perspectives in the UK." British Journal of Ophthalmology 106(10): 1344-1349.

Khasawneh, R. A., S. F. Al-Shatnawi, H. Alhamad and D. Rahhal (2023). "General Public Perceptions and Perceived Barriers Toward the Use of Telehealth: A Cross-Sectional Study from Jordan." Telemedicine journal and e-health : the official journal of the American Telemedicine Association 29(10): 1540-1547.

Kiberu, V. M., R. E. Scott and M. Mars (2019). "Assessing core, e-learning, clinical and technology readiness to integrate telemedicine at public health facilities in Uganda: a health facility - based survey." BMC Health Services Research 19(1): N.PAG-N.PAG.

Kissi, J., B. Dai, C. S. K. Dogbe, J. Banahene and O. Ernest (2020). "Predictive factors of physicians' satisfaction with telemedicine services acceptance." Health Informatics Journal 26(3): 1866-1880.

Klee, D., D. Pyne, J. Kroll, W. James and K. A. Hirko (2023). "Rural patient and provider perceptions of telehealth implemented during the COVID-19 pandemic." BMC health services research 23(1): 981.

Le Bras, A., K. Zarca, M. Mimouni and I. Durand-Zaleski (2023). "Implementing Technologies: Assessment of Telemedicine Experiments in the Paris Region: Reasons for Success or Failure of the Evaluations and of the Deployment of the Projects." International Journal of Environmental Research and Public Health 20(4).

Lee, S., C. E. Begley, R. Morgan, W. Chan and S. Y. Kim (2018). "m-Health Policy Readiness and Enabling Factors: Comparisons of Sub-Saharan Africa and Organization for Economic Cooperation and Development Countries." Telemedicine journal and e-health : the official journal of the American Telemedicine Association 24(11): 908-921.

Li, P., Y. Luo, X. Yu, E. Mason, Z. Zeng, J. Wen, W. Li and M. S. Jalali (2022). "Readiness of healthcare providers for e-hospitals: a cross-sectional analysis in China before the COVID-19 period." BMJ Open 12(2).

Lingg, M. and V. Lütschg (2020). "Health System Stakeholders' Perspective on the Role of Mobile Health and Its Adoption in the Swiss Health System: Qualitative Study." JMIR mHealth and uHealth 8(5): e17315.

Luciano, E., M. A. Mahmood and P. Mansouri Rad (2020). "Telemedicine adoption issues in the United States and Brazil: Perception of healthcare professionals." Health Informatics Journal 26(4): 2344-2361.

Ly, B. A., R. Labonté, I. L. Bourgeault and M. N. Niang (2017). "The individual and contextual determinants of the use of telemedicine: A descriptive study of the perceptions of Senegal’s physicians and telemedicine projects managers." PLoS ONE 12(7).

Ma, Q., D. Sun, Z. Tan, C. Li, X. He, Y. Zhai, L. Wang, F. Cui, M. Li, J. Gao, L. Wang and J. Zhao (2022). "Usage and perceptions of telemedicine among health care professionals in China." International Journal of Medical Informatics 166: N.PAG-N.PAG.

Martin, A. B., J. C. Probst, K. Shah, Z. Chen and D. Garr (2012). "Differences in readiness between rural hospitals and primary care providers for telemedicine adoption and implementation: Findings from a statewide telemedicine survey." Journal of Rural Health 28(1): 8-15.

Mathew, S., M. S. Fitts, Z. Liddle, L. Bourke, N. Campbell, L. Murakami-Gold, D. J. Russell, J. S. Humphreys, E. Mullholand, Y. Zhao, M. P. Jones, J. Boffa, M. Ramjan, A. Tangey, R. Schultz and J. Wakerman (2023). "Telehealth in remote Australia: a supplementary tool or an alternative model of care replacing face-to-face consultations?" BMC health services research 23(1): 341.

Moffatt, J. J. and D. S. Eley (2011). "Barriers to the up-take of telemedicine in Australia--a view from providers." Rural and remote health 11: 1581.

Moroz, I., D. Archibald, D. Archibald, M. Breton, E. Cote-Boileau, L. Crowe, T. Horsley, L. Hyseni, G. Johar, E. Keely, K. K. Burns, C. Kuziemsky, J. Laplante, A. Mihan, A. Mihan, L. Oppenheimer, D. Sturge, D. S. Tuot and C. Liddy (2020). "Key factors for national spread and scale-up of an eConsult innovation." Health Research Policy and Systems 18(1).

Muehlensiepen, F., J. Knitza, W. Marquardt, J. Engler, A. Hueber and M. Welcker (2021). "Acceptance of Telerheumatology by Rheumatologists and General Practitioners in Germany: Nationwide Cross-sectional Survey Study." Journal of Medical Internet Research 23(3): N.PAG-N.PAG.

Murphy, M., L. J. Scott, C. Salisbury, A. Turner, A. Scott, R. Denholm, R. Lewis, G. Iyer, J. Macleod and J. Horwood (2021). "Implementation of remote consulting in UK primary care following the COVID-19 pandemic: A mixed-methods longitudinal study." British Journal of General Practice 71(704): E166-E177.

Nduka, S. O., M. A. Nwaodu and I. J. Nduka (2023). "Telepharmacy Services in a Developing Country: Nigerian Community Pharmacists' and Patients' Perspectives on the Clinical Benefits, Cost, and Challenges." Telemedicine journal and e-health : the official journal of the American Telemedicine Association 29(8): 1238-1251.

Newman, L., N. Bidargaddi and G. Schrader (2016). "Service providers' experiences of using a telehealth network 12 months after digitisation of a large Australian rural mental health service." International Journal of Medical Informatics 94: 8-20.

Nyoni, T., E. C. Evers, M. Pérez, D. B. Jeffe, S. A. Fritz, G. A. Colditz and J. P. Burnham (2023). "Perceived barriers and facilitators to the adoption of telemedicine infectious diseases consultations in southeastern Missouri hospitals." Journal of telemedicine and telecare: 1357633X221149461.

Onsongo, S., C. Kamotho, T. F. Rinke De Wit and K. Lowrie (2023). "Experiences on the Utility and Barriers of Telemedicine in Healthcare Delivery in Kenya." International Journal of Telemedicine and Applications 2023.

Price-Haywood, E. G., C. Arnold, J. Harden-Barrios and T. Davis (2023). "Stop the Divide: Facilitators and Barriers to Uptake of Digital Health Interventions Among Socially Disadvantaged Populations." Ochsner Journal 23(1): 34-42.

Rabinowitz, G., L. D. Cho, N. C. Benda, C. Goytia, K. Andreadis, J. J. Lin, C. Horowitz, R. Kaushal, J. S. Ancker and J. Poeran (2023). "The Telemedicine Experience in Primary Care Practices in the United States: Insights From Practice Leaders." Annals of family medicine 21(3): 207-212.

Ray, K., M. S. Md, K. Felmet, M. Hamilton, M. Md, C. Kuza, R. Saladino, B. Schultz, R. Watson, M. P. H. Scott Md, J. Kahn and M. S. Md (2017). "Clinician Attitudes Toward Adoption of Pediatric Emergency Telemedicine in Rural Hospitals." Pediatr Emerg Care 33(4): 250-257.

Renner, R. M., M. Ennis, A. Kyeremeh, W. V. Norman, S. Dunn, H. Pymar and E. Guilbert (2023). "Telemedicine for First-Trimester Medical Abortion in Canada: Results of a 2019 Survey." Telemedicine and e-Health 29(5): 686-695.

Riew, G. J., F. Lovecchio, D. Samartzis, D. N. Bernstein, E. Y. Underwood, P. K. Louie, N. Germscheid, H. S. An, J. P. Y. Cheung, N. Chutkan, G. M. Mallow, M. H. Neva, F. M. Phillips, D. M. Sciubba, M. El-Sharkawi, M. Valacco, M. H. McCarthy, S. Iyer and M. C. Makhni (2021). "Spine surgeon perceptions of the challenges and benefits of telemedicine: an international study." European Spine Journal 30(8): 2124-2132.

Romero, G., D. de Argila, L. Ferrandiz, M. P. Sánchez, S. Vañó, R. Taberner, P. Pasquali, C. de la Torre, F. Alfageme, J. Malvehy and D. Moreno-Ramírez (2018). "Practice Models in Teledermatology in Spain: Longitudinal Study, 2009-2014." Actas Dermo-Sifiliograficas 109(7): 624-630.

Saigí-Rubió, F., J. Torrent-Sellens and A. Jiménez-Zarco (2014). "Drivers of telemedicine use: comparative evidence from samples of Spanish, Colombian and Bolivian physicians." Implementation Science 9(1): 128-128.

Shi, J., Z. Tan, Q. Ma, D. Sun, Y. Lu, M. ye, L. Wang, F. Cui, X. He, Z. Fan and J. Zhao (2023). "Implementation, service effectiveness and satisfaction with teleconsultation services in China during the COVID-19 pandemic: from the Perspective of Primary Health Care Professionals." International Journal of Medical Informatics 178.

Shiferaw, K. B., S. A. Mengiste, M. K. Gullslett, A. A. Zeleke, B. Tilahun, T. Tebeje, R. Wondimu, S. Desalegn and E. A. Mehari (2021). "Healthcare providers' acceptance of telemedicine and preference of modalities during COVID-19 pandemics in a low-resource setting: An extended UTAUT model." PLoS ONE 16(4 April 2021).

Těšinová, J. K., K. Dobiášová, Z. Dušek and A. Tobiášová (2023). "Development of telemedicine in the Czech Republic from patients' and other key stakeholders' perspective." Frontiers in public health 11: 1202182.

Theis, R. P., J. I. Dorbu, M. E. Mavrodieva, R. A. Guerrero, S. E. Wright, W. T. Donahoo, F. Modave, O. Carrasquillo and E. A. Shenkman (2023). "Telehealth Implementation Response to COVID-19 in the OneFlorida+ Clinical Research Network: Perspectives of Clinicians and Health Systems Leaders." Telemedicine journal and e-health : the official journal of the American Telemedicine Association.

Vakkalanka, J. P., M. M. Nataliansyah, K. A. S. Merchant, L. J. Mack, S. Parsons, N. M. Mohr and M. M. Ward (2022). "Telepsychiatry services across an emergency department network: A mixed methods study of the implementation process." American Journal of Emergency Medicine 59: 79-84.

Vimalananda, V. G., K. Arao, S. Qian, A. Leibowitz, M. F. Zupa, J. Benzer, B. G. Fincke, M. Zocchi, M. Meterko, D. Berlowitz, K. E. Sitter and J. B. Wormwood (2023). "Variation in telehealth use for endocrine care: Patterns and predictors under the “new normal”." Journal of Telemedicine and Telecare.

Wade, V. A., J. A. Eliott and J. E. Hiller (2012). "A qualitative study of ethical, medico-legal and clinical governance matters in Australian telehealth services." Journal of Telemedicine and Telecare 18(2): 109-114.

Walczak, R., M. Kludacz-Alessandri and L. Hawrysz (2022). "Use of Telemedicine Technology among General Practitioners during COVID-19: A Modified Technology Acceptance Model Study in Poland." International Journal of Environmental Research and Public Health 19(17).

Wherton, J., T. Greenhalgh and S. E. Shaw (2021). "Expanding video consultation services at pace and scale in scotland during the covid-19 pandemic: national mixed methods case study." Journal of Medical Internet Research 23(10).

Wubante, S. M., A. M. Nigatu and A. T. Jemere (2022). "Health professionals' readiness and its associated factors to implement Telemedicine system at private hospitals in Amhara region, Ethiopia 2021." PLoS ONE 17(9 September).

Yu-tong, T., Z. Yan, L. Zhen, X. Bing and C. Qing-yun (2022). "Telehealth readiness and its influencing factors among Chinese clinical nurses: A cross-sectional study." Nurse Education in Practice 58.

Zachrison, K. S., K. M. Boggs, E. M. Hayden, J. A. Espinola, C. A. Camargo and C. A. Camargo, Jr. (2020). "Understanding Barriers to Telemedicine Implementation in Rural Emergency Departments." Annals of Emergency Medicine 75(3): 392-399.

Zachrison, K. S., R. E. Cash, K. M. Boggs, E. M. Hayden, A. F. Sullivan and C. A. Camargo (2023). "Emergency Department and Health Care System Factors Associated with Telehealth Innovation During the COVID-19 Pandemic." Telemedicine journal and e-health : the official journal of the American Telemedicine Association.

Zailani, S., M. S. Gilani, D. Nikbin and M. Iranmanesh (2014). "Determinants of telemedicine acceptance in selected public hospitals in Malaysia: Clinical perspective." Journal of Medical Systems 38(9).

Zhang, X. and B. u. Zaman (2020). "Adoption mechanism of telemedicine in underdeveloped country." Health Informatics Journal 26(2): 1088-1103.

Zobair, K. M., L. Sanzogni and K. Sandhu (2019). "Expectations of telemedicine health service adoption in rural Bangladesh." Social Science & Medicine 238: N.PAG-N.PAG.

Zulman, D. M., E. P. Wong, C. Slightam, A. Gregory, J. C. Jacobs, R. Kimerling, D. M. Blonigen, J. Peters and L. Heyworth (2019). "Making connections: Nationwide implementation of video telehealth tablets to address access barriers in veterans." JAMIA Open 2(3): 323-329.
