# Supplementary material for: Enabling Telemedicine From the System-Level Perspective: Scoping Review
Source: J Med Internet Res. 2025 Mar 5;27:e65932. doi: 10.2196/65932 (PMC11923472; doi:10.2196/65932)
Supplement: Multimedia Appendix 3 [file jmir_v27i1e65932_app3.docx]

**Multimedia Appendix 3 Year of publication and number of studies.**

Multimedia Appendix 3 Year of publication and number of studies
